# Necroptosis in acute kidney injury: a shedding light

**DOI:** 10.1038/cddis.2016.37

**Published:** 2016-03-03

**Authors:** S Wang, C Zhang, L Hu, C Yang

**Affiliations:** 1 Department of Urology, Zhongshan Hospital, Fudan University, Shanghai, China; 2 Shanghai Key Laboratory of Organ Transplantation, Shanghai, China; 3 Department of Urology, The First Affiliated Hospital of Soochow University, Suzhou, China; 4 Department of Plastic Surgery, Zhongshan Hospital, Fudan University, Shanghai, China

## Abstract

Acute kidney injury (AKI) is a common and severe clinical condition with a heavy healthy burden around the world. In spite of supportive therapies, the mortality associated with AKI remains high. Our limited understanding of the complex cell death mechanism in the process of AKI impedes the development of desirable therapeutics. Necroptosis is a recently identified novel form of cell death contributing to numerable diseases and tissue damages. Increasing evidence has suggested that necroptosis has an important role in the pathogenesis of various types of AKI. Therefore, we present here the signaling pathways and main regulators of necroptosis that are potential candidate for therapeutic strategies. Moreover, we emphasize on the potential role and corresponding mechanisms of necroptosis in AKI based on recent advances, and also discuss the possible therapeutic regimens based on manipulating necroptosis. Taken together, the progress in this field sheds new light into the prevention and management of AKI in clinical practice.

## Facts

Necroptosis is a kind of regulated necrosis, a novel form of cell death with morphologic features of necrosis but molecularly controlled.
Necroptosis can be widely triggered by death receptors, toll-like receptors, interferons (IFNs), and intracellular protein DNA-dependent activator of IFN regulatory factors in response to viruses. Various upstream signaling converge on mediator receptor-interacting protein kinase 3 and share the common executor mixed lineage kinase domain-like protein.
Necroptosis has been implicated in the pathogenesis of diverse types of AKI to different extents.
Blocking necroptotic pathways by pharmacological inhibitors or genetic manipulation alleviates renal injuries *in vitro* and *in vivo*, indicating a promising outlook in the management of AKI.

## Open Questions

How to monitor necroptosis *in vivo*?
Does necroptosis relate to different stages of AKI? If yes, how?
What is the mechanism of cell-type-specific vulnerability to necroptosis? And what are the clinical implications of this selective sensitivity in AKI?
How does necroptosis interplay with inflammation specifically during AKI?
What is the relative contribution of necroptosis and other forms of regulatory cell death in AKI?

Cell death, serving as an essential process throughout life, has been extensively investigated for decades. Historically, cell death was classified as distinctive forms: apoptosis, autophagic cell death and necrosis.^1^ The term ‘apoptosis', deriving from ancient Greek, has long been recognized synonymously as the programmed cell death, which is developmentally programmed and molecularly controlled. Characteristic morphologic changes of apoptosis include cell shrinkage, nuclear and cytoplasmic condensation, DNA fragmentation, and the formation of apoptosomes.^2^ Autophagic cell death is a newly defined, highly regulated cell death, which is characterized by the markers of autophagy pathway.^3, 4^ In contrast, necrosis was thought to be accidental cell death, and results in cellular swelling, breakdown of plasma membrane integrity and release of intracellular contents, all of which are absolutely distinct from programmed cell death.^5^ Cell death, no matter which specific form, has a key role in maintaining tissue homeostasis as well as other crucial biological processes.

However, the full extent of cell death has not been explored. Recent breakthroughs in this field shed light on regulated necrosis, a new type of cell death with morphologic features of necrosis but genetically determined.^6^ It has been well-documented that necroptosis is highly orchestrated regulated necrosis dependent on receptor-interacting protein kinase 3 (RIP3) and mixed lineage kinase domain-like protein (MLKL).^7, 8^ It not only involves in the development of organism but also participates in various pathophysiological processes. An accumulating body of evidence has demonstrated that necroptosis contributes to the pathogenesis of numerous diseases and tissue damages, including ischemic brain injury, myocardial infarction, pancreatitis as well as liver and renal injury.^8^

Acute kidney injury (AKI) is still a critical problem in clinical practice, with a heavy health burden around the world.^9^ AKI affects ~13.3 million individuals and contributes to ~1.7 million deaths globally per year.^10^ Apoptosis was recognized as the main form of cell death that is responsible for renal dysfunction in AKI.^11^ Therefore, for a long time, strategies targeting the apoptosis pathway have been widely explored for AKI treatment. Despite the substantial therapeutic effect in animal models, the efficient anti-apoptosis intervention strategies are still absent in clinic. This could be partly ascribed to our limited understanding of the complex cell death mechanism in the process of AKI. Recent progress suggested that besides apoptosis, other forms of cell death, including regulated necrosis (such as, necroptosis, necrosis by mitochondrial permeability transition, pyroptosis, parthanatos and ferroptosis), autophagic cell death and mitotic catastrophe, also significantly contribute to the pathogenesis of AKI.^12, 13^ As the best-characterized form of regulated necrosis, necroptosis has been intensively explored in the setting of AKI. Understanding the precise role of necroptosis in AKI might provide potential therapeutic regimens. Therefore, this review summarized the potential role and corresponding mechanisms of necroptosis in AKI based on recent advances, and also discussed the possible therapeutic strategies based on manipulating necroptotic pathways.

## The signaling pathways of necroptosis

**Figure 1:**
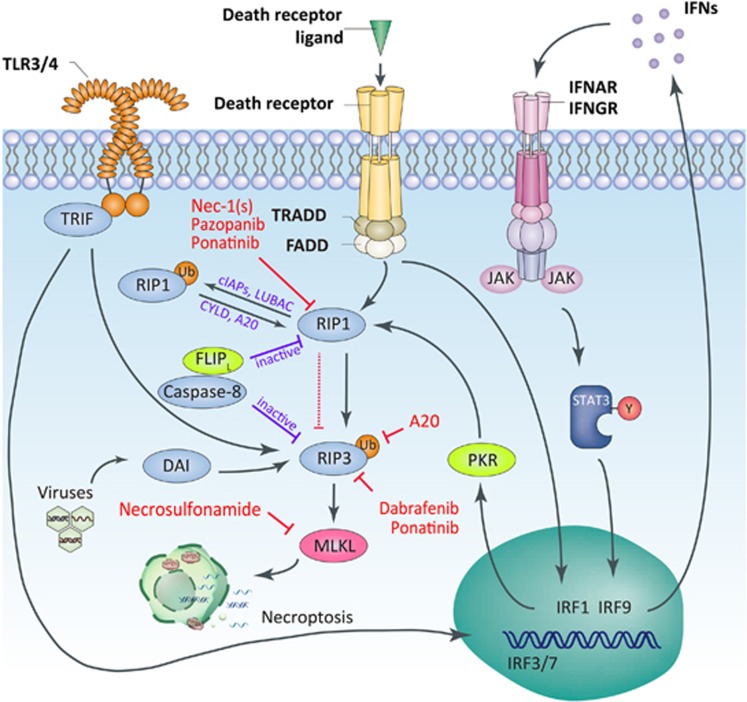
Schematic overview of necroptotic pathway. Necroptosis is triggered by various stimuli, including engagement of death receptors and Toll-like receptors, IFNs and intracellular protein DAI in response to viruses. Diverse upstream signals converge on mediator RIP3, and consequently activate the executor MLKL. Especially, an autocrine loop via *de novo*-transcribed IFNs/IFNAR1 signaling is believed to play a crucial role in sustaining the activation of necroptosis. Physiological regulators or pharmacological inhibitors can regulate necroptotic pathway at different molecular levels. Necrosulfonamide only acts on human MLKL

As the identification of necrostatins as specific inhibitors targeting receptor-interacting protein kinase 1 (RIP1), the signaling pathways of necroptosis have been extensively determined.^14, 15^ Engagement of death receptors and Toll-like receptors (TLRs), interferon (IFN) signals, as well as intracellular stimuli from protein DNA-dependent activator of IFN regulatory factors (DAI) in response to viruses are able to initiate necroptosis^8^ (Figure 1). Among them, tumor necrosis factor (TNF)-α-induced necroptosis in the presence of caspase inhibition is the best-characterized model. After TNF-α binds to TNF receptor (TNFR)1, the adaptor molecules Fas-associated death domain (FADD) and TNF-receptor-associated death domain recruit RIP1 that subsequently combines with RIP3^16, 17, 18^ to form a complex termed ‘necrosome'.^19^ It is believed that the oligomerization driven by the RIP homotypic interaction motif (RHIM) domain on RIP3 and RIP1 leads to the autophosphorylation of RIP3, which results in the activation of RIP3.^17, 18^ In accordance, other initial participants like Toll/IL-1 receptor domain-containing adaptor inducing IFN-β (TRIF) and DAI use the RHIM domain to activate RIP3, indicating that RHIM domain has an important role in triggering necroptosis. Activated RIP3 recruits and phosphorylates the downstream MLKL, which serve as the executor of necroptosis.^20, 21^ It is demonstrated that phosphorylation of MLKL induces a molecular switch that enable MLKL to translocate to the membrane and consequently disrupt the plasma membrane. However, the exact mechanism needs to be further illustrated.^7, 22^ Moreover, it seems that MLKL could cause mitochondria fission via phosphoglycerate mutase family member 5 and dynamin-related protein 1.^23^ Whereas, the depletion of mitochondria has been determined not to prevent necroptosis in another study, suggesting that the mitochondrial fragmentation by MLKL may contribute little to the final execution of necroptosis.^24^

Other factors beyond TNF-α could also initiate RIP3-dependent necroptosis. It has been reported that activation of TLR3 or TLR4 by polyinosine–polycytidylic acid (poly(I:C)) and lipopolysaccharide (LPS), respectively, in the presence of zVAD (a pan-caspase inhibitor) leads to necroptosis through the adaptor protein TRIF, which contains RHIM domain to interact with RIP3.^25, 26^ Additionally, both type I (α/β) and type II (γ) IFNs were demonstrated to trigger necroptosis when FADD and caspase-8 are absent. IFNs-induced necroptosis proceeds by upregulating the expression of protein kinase R (PKR), which relies on JAK-STAT pathway. The PKR interacts with RIP1 and promotes the formation of the ‘PKR necrosome' consisting of PKR, RIP1 and RIP3 to implement necroptosis.^27^ It should be noted that type I IFNs have been recently shown to have a predominant role in sustaining the activation of the RIP1–RIP3 complex.^28^ Macrophage deficient in IFN-α receptor (IFNAR) 1 was found to be resistant to LPS–TLR4-, poly(I:C)–TLR3- and TNF–TNFR1-mediated necroptosis in the presence of caspase inhibitors. Furthermore, it is proposed that LPS-, poly(I:C)-, TNF- and IFN-β-induced necroptosis in macrophages proceed through IFN regulatory factors-dependent *de novo* transcription of IFNs to facilitate the activation of necrosome. Also, the autocrine loops via *de novo* IFNs/IFNAR1 amplifies the initiating signals of necroptosis. Therefore, it is likely that type I IFNs provide a positive feedback to license the final execution of necroptosis. However, the ‘license' role of type I IFNs in other cells need to be further validated.

Besides extrinsic pathways, intracellular signaling can also lead to necroptotic cell death. Viral infection-induced expression of cytosolic DAI could interact with RIP3 by RHIM domain and forms DAI–RIP3 complex with initiating function analogous to the RIP1–RIP3 necrosome or TRIF–RIP3 complex.^29^

These studies indicate that there exist diverse upstream pathways dependent on different stimuli. Various upstream signals converge on the RIP3 and share the same downstream executing pathway.

## Key regulators of necroptosis

### RIP1

Why *Rip1*-deficient mice die perinatally remains an intriguing question for a long time. Recent exciting findings showed that kinase-inactive *Rip1* mutant D138N and K45A knockin mice are viable.^30, 31^ Importantly, in addition, cells derived from these mice are resistant to necroptosis, but mediate NF-κB pathway normally. These studies indicate that the kinase activity of RIP1 is indispensable and might serve as a ‘permission switch' in the necroptotic pathways. Ironically, on the other hand, RIP1 itself might act as an inhibitor of necroptosis when its kinase activity is functionally absent. There probably exists an underlying physiological mechanism regulating the ‘permission switch' of RIP1, thereby providing a negative feedback loop to restrict the magnitude of necroptosis. According to this theory, loss of RIP1 could result in the overreaction of necroptosis that might account for the lethality of *Rip1*-knockout mice. Consistently, *Rip1*^*−/−*^ cells have high sensitivity to necroptotic stimuli.^32^ However, the proposed inhibitory effect of RIP1 needs further validation.

### cIAPs, LUBAC and CYLD

In fact, not only necroptosis but also apoptosis and NF-κB pathways can be triggered by the engagement of TNFR1. During this process, cellular inhibitor of apoptosis proteins (cIAPs), linear ubiquitin chain assembly complex (LUBAC) and cylindromatosis (CYLD) are reported to have crucial roles in deciding the switch between different cellular outcomes.^33, 34, 35, 36, 37^ Briefly, TNFR1 signaling leads to the formation of distinct types of complexes with different functions. Polyubiquitination of RIP1 by cIAPs enables recruitment of LUBAC, which in turn stabilizes a so-called prosurvival complex (complex I) by generating the linear ubiquitin chains on RIP1. Subsequently, complex I leads to the well-known NF-κB signaling. Conversely, deubiquitination of RIP1 by CYLD or the absence of cIAPs and LUBAC renders complex I unstable and facilitates other complexes assembled to initiate apoptosis or necroptosis.

### Complex of RIP1, FADD, caspase-8 and cFLIP isoforms

When cIAPs are absent, RIP1, FADD, caspase-8/10 and FADD-like interleukin (IL)-1β-converting enzyme (FLICE)-inhibitory protein (cFLIP) isoforms assemble an intracellular complex referred to as ripoptosome.^38^ Within the complex, cFLIP forms heterodimer with caspase-8, and controls the caspase activity. Depending on the isoforms of cFLIP, ripoptosome could lead to either apoptosis or necroptosis.^38, 39, 40^ cFLIP_L_ (the long isoform of cFLIP)-caspase-8 heterodimer has restricted enzymatic activity that could inactivate RIP1 and RIP3 through cleavage, and consequently inhibits necroptosis and favors apoptosis; conversely, the heterodimer of caspase-8 and cFLIP_S_ (the short isoform of cFLIP) lacks such catalytic activity and sensitizes cells to TLR- and Fas-induced necroptosis.^38, 41^ In the absence of cIAPs, RIP1 dissociates from complex I and forms an assembly consisting of RIP1, FADD, caspase-8 and long isoform of FADD-like interleukin (IL)-1β- converting enzyme (FLICE)-inhibitory protein (FLIP_L_), which favors apoptosis and inhibits spontaneous necroptosis. Consistently, *Caspase-8*-deficient mice die as embryos with heart defects,^42^ and this embryonic lethality can be rescued by *Rip3* deletion.^43^ This phenomenon implies the negatively regulatory role of caspase-8 in necroptosis pathways. Of note, caspase-8 seems to inhibit necroptosis by forming a heterodimer with FLIP_L._^39, 40^ High-expression level of FLIP_L_ facilitates the formation of caspase-8–FLIP_L_ complex, which cleaves and inactivates RIP1. Moreover, embryonic death of *Fadd*-deficient mice can also be prevented by *Rip3* deletion, indicating that FADD also plays an inhibitory role.^44, 45^ Complex of RIP1, FADD, caspase-8 and FLIP_L_ will divert to necrosome and necroptosis will be induced when caspase-8-FLIP_L_ heterodimer is functionally defective or FADD is absent.

### A20

RIP3 is required to be ubiquitinated at Lys5 to facilitate the formation of necrosome during necroptosis, while A20 could inhibit necroptosis by targeting this process.^46^ This theory is further validated by the phenomenon that loss of *Rip3* reverses the lethality of *A20*-deficient mice.^46^

## Necroptosis in AKI: evidence and implications

**Figure 2:**
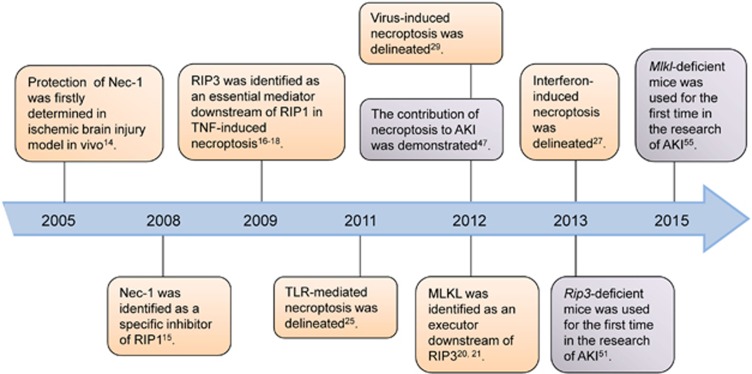
Timeline for major events in the studies of necroptosis and its role in AKI. Orange boxes represent the findings in the discovery of necroptotic pathway; purple boxes demonstrate the breakthroughs in researches of necroptosis in AKI

As shown in Figure 2, the presence of necroptosis in AKI was first determined in a murine model of renal ischemia–reperfusion injury (IRI) by Linkermann *et al.*^47^ who evaluated the protective effect of necrostatin-1 (Nec-1), a chemical inhibitor of RIP1. Nec-1 was shown to significantly alleviate the renal dysfunction and tissue damage, supporting the vital role of necroptosis in the pathogenesis of ischemic AKI. Surprisingly, compared with Nec-1, treatment with the pan-caspase inhibitor zVAD to block apoptosis did not provide any detectable protection. This finding contradicts with a previous report that demonstrated the protective effect of zVAD in the context of IRI.^48^ One possible reason is the incontinence in the clamping time of the renal pedicles adopted in these studies, which may result in diverse profiles of cellular death in kidneys. Besides, the different timing of zVAD administration may be another influence. It is known that apoptosis does not occur immediately after the onset of ischemia.^11^ Thus, it cannot be ruled out the possibility that application of zVAD just 15 min before ischemia might diminish its therapeutic effect. Both factors could be partially responsible for the ‘ineffectiveness' of zVAD observed in this research. The role of necroptosis in ischemic kidney injury was further suggested in subsequent studies. Consistently, two researches confirmed the protection of Nec-1 in rat and human renal tubular epithelia cells (TECs), respectively, in a model of TNF-α stimulation and ATP depletion that mimics the renal ischemic injury *in vitro.*^49, 50^ Furthermore, genetic model provided more convincing evidence of necroptosis in renal IRI. Protection from ischemic damage was exhibited in *Rip3*-knockout mice, and further amelioration by addictive Nec-1 administration was not observed, implicating that *Rip3*-dependent necroptosis pathway participates in ischemic AKI as a crucial mediator.^51^ *Rip3*-deficient mice demonstrate a normal phenotype except for a slight failure to gain weight.^52, 53^ Notably, on the other hand, *Rip3*-knockout mice might also exert addictive protection due to its obviously increased peritubular diameters than wild-type mice.^53^ Therefore, tissue-specific *Rip3*-knockout models are needed to provide more explicit evidence. Taken together, these findings suggested that necroptosis is of crucial importance for the pathologic processes of renal IRI and targeting necroptotic pathway molecular emerges as a promising treatment option to improve the prognosis of ischemic AKI. However, the mechanism by which necroptosis contributes to renal IRI has not be fully delineated, which invites further investigations.

In addition to renal ischemic injury, necroptosis also contributes to AKI induced by cisplatin, a widely used chemotherapy agent with nephrotoxic effect. Tristao *et al.*^54^ found that zVAD prevents the cisplatin-associated damage on human renal TECs *in vitro* and combined use of zVAD and Nec-1 can provide additional protection. In agreement, Linkermann *et al.*^51^ showed an evident protection in *Rip3*-knockout mice in the background of cisplatin-induced AKI. Importantly, a more recent study by Xu *et al.*^55^ used *Mlkl*-knockout mice to investigate the role of necroptosis in AKI for the first time, providing more reliable evidence due to the indispensable role of MLKL in necroptotic pathway. Notably, it is shown that the increased production of TNF-α, TNF-related weak inducer of apoptosis (TWEAK) and IFN-γ (also known as TTI) in cisplatin-induced AKI may be an important promoter of necroptosis.^55^ However, whether TTI induces necroptosis of renal tissue *in vivo* directly in the background of cisplatin-associated AKI was not answered in this research. The nephrotoxicity of cisplatin remains a severe obstacle in its clinical application as an anticancer agent. One of the characteristics of cisplatin-induced AKI is the upregulation of proinflammatory cytokines.^56^ But to what extent these proinflammatory cytokines are related to necroptosis activation is unknown. More researches to address this question are urgently needed, which can optimize a combined therapeutic strategy to improve the efficacy of blocking necroptotic signaling.

Likewise, blocking necroptosis pathway by chemical inhibitor Nec-1 or gene knockout of key mediators can protect from AKI initiated by other nephrotoxic agents. For example, cyclosporin A (CsA) is a widely used immunosuppressive drug for organ transplantation and other autoimmune diseases. Necroptosis was also suggested in CsA-associated tubular injury with an *in vitro* model.^57^ The authors demonstrated obvious therapeutic effects of Nec-1 and knockdown of *Rip3* in rat TECs exposed to CsA, indicating that necroptosis might also be implicated in the pathologic process of CsA-related AKI. Furthermore, Nec-1 was similarly shown to prevent from contrast-induced AKI in another study.^58^ Unfortunately, Nec-1 showed no relevance with cell death in this model but an unexpected effect on renal peritubular diameters. Thus, it cannot be excluded that the protection of Nec-1 is due to affecting renal blood flow but not blocking necroptosis pathways in this setting.

Recently, the contribution of necroptosis to AKI was determined in a glycerol-induced rhabdomyolysis model.^59^ The authors identified necroptosis, which is mainly mediated by increased circulating TNF, as the predominant form of tubular injury in this background. Meanwhile, cardiac injury was also observed in this model. Oppositely, necroptosis was detected to be of minor importance and apoptosis acts as a crucial mediator in the cardiac damage during rhabdomyolysis. This study exemplified the difference of organ-specific susceptibility to necroptotic cell death in the same circumstance. The overexpression of FLIP in kidneys may help drive the TNF signaling pathway towards necroptosis, but the exact mechanism remains to be illustrated.

Collectively, accumulating evidence has been extensively reported in recent years to demonstrate that necroptosis plays an crucial role in different types of AKI, suggesting a potential therapeutic target for this medical condition that needs to be clinically validated in the future (Tables 1 and 2).

## Open questions

### How to monitor necroptosis *in vivo*?

Substantial progress made in the past decade brought insights into the molecular mechanism of necroptosis, as well as its contribution to the pathogenesis of AKI. Until now, however, these studies have confined to preclinical models, and the diagnosis of necroptosis has still largely relied on detecting the protective effect of Nec-1 or knockout of *Rip3* and *Mlkl*. However, protection of Nec-1 alone cannot provide a definite identification of necroptosis as its nonspecific functions on renal peritubular diameters, indolamin-2, 3-dioxygenase (IDO) and ferroptosis;^58, 60, 61^ and one, therefore, should be cautious when the presence of necroptosis is based only on the effect of Nec-1. Exploring the role of necroptosis *in vivo* requires reliable and feasible detection approaches. Antibodies that target phosphorylated MLKL through immunostaining or immunoblotting methods emerge as promising biomarkers to directly detect necroptosis. RIP3 could phosphorylate the activation loop of murine MLKL at Ser345, Ser347 and Thr349.^62^ A recent study from Rodriguez *et al.*^63^ indicated that phosphorylation of Ser345 is critical for RIP3-mediated necroptosis, during both the processes of MLKL activation and execution. They further generated a specific monoclonal antibody to detect phospho-Ser345 in murine cells.^63^ Distinctly, RIP3 phosphorylates human MLKL at the Thr357 and Ser358 sites.^21^ Wang *et al.*^64^ found that phosphorylation of both sites are essential for the engagement of necroptosis, and developed a monoclonal antibody that specifically recognizes human phospho-MLKL. This antibody has been applied to detect necroptosis *in situ* based on human liver biopsy samples from patients with drug-induced liver injury,^64^ although further validation in other disease models is required before its clinical application. To measure specific phosphorylation site of MLKL, other than to detect expression levels of pathway molecules, seems more efficient and accurate. It is inspired that commercial mAbs against murine and human phosphorylated MLKL are available now. Moreover, besides biopsy samples, biomarkers derived from blood or urine may also promise to offer clinically feasible tools to evaluate the dynamics of necroptosis *in vivo*. For instance, measurement of graft-derived circulating cell-free DNA has shown promise as a way to detect severe graft injury using droplet digital PCR.^65^ Hence, development of specific biomarkers is urgently needed in the future.

### Necroptosis in AKI: when and where?

Some basic questions about necroptosis in AKI remain to be addressed. For example, whether and how necroptosis relates to different stages of AKI are still unknown. AKI consists of several mutually overlapping stages: initiation, extension, maintenance and recovery phases. Necroptosis may contribute to a variable degree to tissue injury and regeneration in various phases. Understanding this question enables better therapeutic strategies for AKI targeting at necroptosis. Recent knowledge recognizes necroptosis as a crucial participant in the early stage of AKI. In a previous study, furthermore, extra doses of the Nec-1 at 2 and 4 h after renal reperfusion followed ischemia that demonstrated no addictive protective effect.^47^ Despite of this observation, it cannot be concluded that necroptosis occurs in a snapshot fashion. The time course of necroptosis in various types of AKI is worthy of being further delineated.

Another question involves in the cell-specific sensitivity to necroptosis within kidney and its underlying mechanism. Compared with mesangial cells and podocytes, glomerular endothelial cells and tubular cell are more susceptible to TNF-triggered necroptosis *in vitro.*^47^ It is likely the expression level of RIP3 could serve as a predictor for cellular sensitivity towards necroptosis, whereas the exact regulating mechanism remains unclear. Understanding the precise susceptibility profiles of renal cells could help develop more efficient treatment aiming at certain cell types that are more vulnerable to necroptosis. More researches are required and tissue-specific gene deletion model should be used for better investigation of exact role of necroptosis in various renal cells during AKI.

### How does necroptosis interplay with inflammation specifically during AKI?

Unlike apoptosis, necroptosis results in the release of unprocessed intracellular contents, also known as damage-associated molecular pattern (DAMP).^22, 66^ Subsequently, DAMP activates the innate immunity to produce more proinflammatory cytokines, which in turn leads to more necrosis in renal tissues. This autoamplification loop of inflammation is termed as necroinflammation;^67, 68^ and the conceptual advance very well delineates the process of immune cascades induced by necroptosis as well as other necrotic cell deaths. Specially, it is reported that necroptotic cells are characterized with increasing secretory IL-33.^69^ IL-33 is a pleiotropic cytokine of the IL-1 family and has an important role in tissue homeostasis.^70^ Increased level of extracellular IL-33 indicated that necroptosis might not be such proinflammatory as originally assumed. A recent study showed that liver-resident macrophages experience necroptosis during bacterial infection.^71^ Considering the abundance of tissue-resident immune cells within the kidney,^72, 73, 74^ whether they occur necroptosis upon various initiating stimuli remains an intriguing question. Furthermore, how does necroptosis orchestrate the dynamics of both resident and infiltrating immune cells? And how necroptosis is connected with the phased transition of immune cells from destructive to reparative role during AKI? All these questions need more studies in depth in the future.

### What is the relative contribution of necroptosis and apoptosis in AKI?

Undoubtedly, necroptosis and apoptosis coexist in the pathophysiological process of AKI. However, whether necroptosis is relevant to the damage of kidney function during AKI are challenged recently. Despite some limitations in the detection techniques for apoptosis,^11, 75^ the effectiveness of anti-apoptosis therapeutic interventions in previous studies have proven the contribution of apoptosis to AKI,^11^ which should not be neglected even from current perspective. In fact, necroptosis, apoptosis and other modes of regulated cell death orchestrate the pathogenesis of AKI together; and the relative contribution of each cell death to AKI depends on the type and severity of the injury. The relative contribution of each one to the renal dysfunction in AKI remains elusive, and therefore exploring in more detail the contribution of necroptosis, apoptosis as well as other types of cell death in certain conditions of AKI will optimize specific therapeutic modalities.

## Therapeutic implications

Identification of chemicals inhibiting the critical checkpoints makes it possible to manipulate necroptotic pathway. Nec-1, originally identified as the RIP1 inhibitor, has been widely used to treat necroptosis-associated diseases. However, a recent study has observed that Nec-1 could protect *Rip1*^−*/*−^ cells from ferroptosis, indicating potential off-target effects of Nec-1 on ferroptosis.^61^ Besides, Nec-1 has yet unrecognized effects on renal peritubular diameters as mentioned above,^58^ and an unexpected inhibitory role on IDO.^60^ All these nonspecific functions of Nec-1 as well as the relatively short half-life period^60^ hamper its final clinical application. In spite of possible similar pharmacokinetic properties, a more-specific variant Nec-1 s that does not affect either ferroptosis or IDO provides a better alternative.^60, 61^ Beyond Nec-1(s), a series of necroptosis inhibitors have been reported recently.^21, 26, 63, 76, 77, 78, 79, 80, 81^ Particularly, two independent groups performed screens with a range of FDA-approved agents and identified three of them as potential drugs to block necroptosis. Dabrafenib was recognized as a selective inhibitor for RIP3,^79^ pazopanib for RIP1, and surprisingly, ponatinib for both RIP1 and RIP3.^76^ Given that the toxicities of pharmacological candidates are critical considerations in the clinical application, these drugs are more advantageous regarding this issue, for all of them are anticancer agents in current clinical use with well-documented side effects. However, whether such side effects are specifically acceptable for patients with necroptosis-associated diseases remains another question that warrants further elaborate evaluation. Considering the multiple initiating pathways at upstream levels, manipulating downstream mediators of necroptosis such as MLKL may be more effective. A direct inhibitor of human MLKL, necrosulfonamide, holds the promise to serve as a therapeutic drug.^21^ However, as many inhibitors interfering necroptosis have not been extensively explored to date, the efficacy and safety of these potential drugs should be further carefully validated.

In addition to inhibitors of key mediators, it is also possible to block necroptosis at the level of receptors. But the contribution of each receptor to differently induced AKI is uncertain, which complicates the development of a feasible and effective therapy. Thus the role of different receptors in various type of AKI must be clearly described in the future. Moreover, understanding the exact executive mechanism in the downstream of MLKL might lead to novel potential therapeutic checkpoints.

One should be cautious about the unwanted effect of necroptosis blockade on other forms of cell death or vice versa. For example, molecular pathways of necroptosis and apoptosis could crosstalk at various levels and therefore could mutually impact each other. This theory can be exemplified by zVAD that is shown to shift apoptosis to necroptosis.^51^ Therefore, more preclinical animal model researches are needed to clarify any possible reciprocal effects between necroptosis and other forms of cell death.

Theoretically, blocking necroptosis not only ameliorates cell death, but also reduces release of DAMPs as well as the subsequent inflammation, thus providing a promising therapeutic option. However, no necroptosis inhibitors are presently used for treatment of necroptosis-associated diseases in clinic. Although necroptosis inhibitors have entered clinical trials, several considerations should be seriously taken into account. Current data supported that an early intervention might exert a satisfying therapeutic effects, which is also implied by the necroinflammation theory. Unfortunately, such strategy might not be practical in a large fraction of cases, for AKI is usually asymptomatic at the early stage. This is why the time course of necroptosis discussed previously in the ‘open question' section is particularly important. Because necroptosis can hardly be transient in AKI, the therapeutic window might be wide enough, which of course requires further validation. The establishment of such clinical trials in patients who have some certain predictable risk factors for AKI may be much easier, for example, kidney transplantation recipients. Some iatrogenic factors can also cause AKI, such as cardiac surgery. AKI has high incidence under these conditions, particularly among those with preexisting renal comorbidities (high-risk patients).^82, 83^ Therefore, prophylactic strategies are recommended under these situations. Meanwhile, the prophylactic strategy and early-onset intervention could be used in combination on a case-by-case basis, because the intensive management for these patients provides better detection for AKI in the early phase. It is reported that recipient mice receiving kidneys from *Rip3*^*-/-*^ donors have longer survival and improved renal function.^84^ However, in clinic, it is not ethically or practically feasible to treat either living or deceased donors in kidney transplantation. Fortunately, cold ischemia time offers an ideal therapeutic window for preconditioning allografts with necroptosis inhibitors *ex vivo*. The trial designers should be precautious about the possible synergistic effects of necroptosis blockage and immunosuppression on acute severe infection.

A safety concern related to the increased risk of viral infection should be specially considered in the establishment of ‘anti-necroptosis' clinical trials. The prevailing theory believes that necroptosis is a defensive mechanism against virus in physiological conditions.^85^ As a classic example, *Rip3*-deficient mice died during vaccinia virus infection.^18^ Although these are no sufficient evidence indicating that necroptosis is crucial in the control of whole spectrum of viruses and pharmacological inhibitors of necroptosis could result in viral infection, the safety concern regarding this problem remains a critical issue. Information on this potential risk should be included in the informed consent processes for participants in the clinical trials. In addition, careful screening for potential infections should be employed during anti-necroptosis treatment, especially for the recipients of renal transplantation.

Finally, given the fact that there are various and complex mechanisms contributing to AKI pathogenesis, it is reasonable to adopt a combination strategy. Blocking necroptosis in conjunction with current clinical treatment can be applied to improve the therapeutic efficacy and reduce side effects.

## Conclusions

Taken together, necroptosis has been suggested to serve as a crucial role in AKI. The improving understanding of the underlying mechanism of necroptosis reveals a novel ‘checkpoint' for AKI treatment. It is inspired that necroptosis inhibitors have entered clinical trials. And blocking necroptosis holds the great promise to improve the prophylaxis and prognosis of AKI in the clinical practice.

## Figures and Tables

**Table 1:** Summary of necroptosis in different AKI models *in vitro*

Year	Cell line/type	Induction	Therapeutic intervention	Effect of zVAD	Reference
2012	Mice tubular cell line TKPTS, mice glomerular endothelial cell line glENDp54	TNF-α/cycloheximide in the presence of zVAD	Nec-1; *Rip1* siRNA; *Rip3* siRNA	NA	47
2012	Human proximal tubular cell HK-2	Cisplatin	Nec-1; Nec-1+zVAD	Protective	54
2012	Rat tubular cell line NRK-52E	CsA	Nec-1; *Rip3* siRNA	NA	57
2013	Rat tubular cell line NRK-52E	TNF-α/antimycin A	Nec-1	NA	49
2013	Mice renal proximal tubular cells	TNF-α/TWEAK/IFN-γ	Nec-1	Harmful	51
2014	Human proximal tubular cell HK-2	TNF-α/antimycin A in the presence of zVAD	Nec-1	NA	50
2015	Mice renal proximal tubular cells	Cisplatin	Nec-1; *Rip3*-KO; *Mlkl*-KO; *Rip1* shRNA	No effect	55

Abbreviations: CsA, cyclosporin A; IFN, interferon; TNF, tumor necrosis factor; TWEAK, TNF-related weak inducer of apoptosis.

**Table 2:** Summary of necroptosis in different AKI models *in vivo*

Year	Model	Possible initiating molecules	Therapeutic intervention	Effect of zVAD	Reference
2012	Renal ischemia/reperfusion injury	NA	Nec-1	No effect	47
2013	Renal ischemia/reperfusion injury; cisplatin-induced AKI	NA	Nec-1; *Rip3*-KO	Protective in cisplatin-induced AKI	51
2013	Contrast-induced AKI	NA	Nec-1	No effect	58
2015	Cisplatin-induced AKI	TNF-α, TWEAK, IFN-γ	Nec-1; *Rip3*-KO; *Mlkl*-KO	NA	55
2015	Cardiorenal injury after glycerol-induced rhabdomyolysis	TNF-α	Infliximab (TNF-α antibody); Nec-1	NA	59

Abbreviations: AKI, acute kidney injury; IFN, interferon; Nec-1, necrostatin-1; TNF, tumor necrosis factor; TWEAK, TNF-related weak inducer of apoptosis.

## Abbreviations

AKI: acute kidney injury
CsA: cyclosporin A
CYLD: cylindromatosis
DAI: DNA-dependent activator of IFN regulatory factors
DAMP: damage-associated molecular pattern
FADD: Fas-associated death domain
cFLIP: cellular FADD-like interleukin (IL)-1β- converting enzyme (FLICE)-inhibitory protein
cIAPs: cellular inhibitor of apoptosis proteins
IDO: indolamin-2, 3-dioxygenase
IFN: interferon
IFNAR: IFN-α receptor
IRI: ischemia–reperfusion injury
LPS: lipopolysaccharide
LUBAC: linear ubiquitin chain assembly complex
MLKL: mixed lineage kinase domain-like protein
Nec-1: necrostatin-1
poly(I:C): polyinosine–polycytidylic acid
PKR: protein kinase R
RHIM: RIP homotypic interaction motif
RIP1: receptor-interacting protein kinase 1
RIP3: receptor-interacting protein kinase 3
TLR: toll-like receptor
TNF: tumor necrosis factor
TNFR: TNF receptor
TWEAK: TNF-related weak inducer of apoptosis
TEC: tubular epithelia cell
